# Electrochemiluminescence in paired signal electrode (ECLipse) enables modular and scalable biosensing

**DOI:** 10.1126/sciadv.abq4022

**Published:** 2022-09-21

**Authors:** Young Kwan Cho, Hyunho Kim, Alan Bénard, Hyun-Kyung Woo, Franziska Czubayko, Paul David, Frederik J. Hansen, Jong Ik Lee, Jay Hoon Park, Emmanuel Schneck, Georg F. Weber, Ik-Soo Shin, Hakho Lee

**Affiliations:** ^1^ Center for Systems Biology, Massachusetts General Hospital, Boston, MA 02114, USA.; ^2^ Department of Chemistry, University of Massachusetts Lowell, Lowell, MA 01854, USA.; ^3^ Department of Radiology, Harvard Medical School, Boston, MA 02115 USA.; ^4^ Department of Surgery, Friedrich-Alexander University (FAU) Erlangen-Nürnberg, Universitätsklinikum Erlangen, Erlangen, Germany.; ^5^ Department of Chemical and Biomolecular Engineering, Sogang University, Seoul 04107, Republic of Korea.; ^6^ Department of Plastics Engineering, University of Massachusetts Lowell, Lowell, MA 01854, USA.; ^7^ Department of Anesthesiology, Operative Intensive Care Medicine and Pain Therapy, Justus Liebig University of Giessen, Rudolf-Buchheim-Strasse 7, 35392 Giessen, Germany.; ^8^ German Centre for Infection Research (DZIF), Partner Site Giessen-Marburg-Langen, 35392 Giessen, Germany.; ^9^ Department of Chemistry, Soongsil University, Seoul 06978, Republic of Korea.

## Abstract

Electrochemiluminescence (ECL) has an inherently low background and enables precise chemical reactions through electrical control. Here, we report an advanced ECL system, termed ECLipse (ECL in paired signal electrode). We physically separated ECL generation from target detection: These two processes were carried out in isolated chambers and coupled through an electrode. The strategy allowed us to minimize cross-chemical reactions, design electrodes for high ECL signals, and integrate multiple sensors in a chip. As a proof of concept, we implemented an eight-plex ECLipse and applied it to detect host factors in human plasma. ECLipse achieved higher signal-to-noise ratio than conventional ECL assays and was >7000-fold more sensitive than enzyme-linked immunosorbent assay. In a pilot clinical study, we could detect septic conditions by measuring host factors [i.e., interleukin-3 (IL-3), IL-6, and procalcitonin (PCT)]. ECLipse assay further revealed distinct IL-3 and IL-6 patterns in patients with severe acute respiratory syndrome coronavirus 2 infection.

## Teaser

Electrochemiluminescence in an advanced electrode system enabled exquisitely sensitive immunoassays for sepsis diagnostics.

## INTRODUCTION

Cytokines (CKs) are emerging biomarkers in health and disease. CKs are potent small proteins that modulate host immune systems in response to disease; monitoring CK profiles in accessible bodily fluids (e.g., blood and urine) can inform diagnostics during infection, sepsis (*1*), trauma, and cancer (*2*, *3*). Translating CK assays into clinical settings, however, faces practical challenges. Ideal CK tests should detect multiple protein targets below femtomolar ranges, given that CKs usually are present at very low concentrations (*4*). The tests also need to be fast and carried out near the bedside to help clinicians initiate timely treatment for acute diseases (e.g., CK storms) (*5*). Unfortunately, the current gold standard, enzyme-linked immunosorbent assay (ELISA), often fails to meet these requirements (i.e., sensitivity, speed, and point-of-care use), underscoring the need for an alternative approach.

Electrochemiluminescence (ECL) is a powerful biosensing technique (*6*–*8*). The method is based on a signaling reaction (chemiluminescence) that can be triggered by electrical potential. This unique turn-on mechanism enables ECL assays to (i) achieve exquisite sensitivity with negligible optical background, (ii) sync the reaction timing to generate a consistent analytical signal, and (iii) afford measurement setups simpler than fluorescent or electrochemical (EC) detection (*9*, *10*). These technical merits led to the development of various ECL biosensors, including commercial ones (*9*, *11*). Most ECL systems, however, adopt the same format of ELISA or chemiluminescence assays: Biorecognition and light generation occur in one reaction chamber. Although straightforward to execute, this one-pot scheme carries the risk of cross-reactions among reagents (e.g., biomolecules, luminophores, and coreactants), limits assay optimization, and underuses ECL’s electrical controllability.

Here, we report a modular, scalable strategy for highly sensitive ECL assays, termed ECLipse (ECL in paired signal electrode). Adopting the concept of bipolar electrode (BPE) (*9*, *12*, *13*), ECLipse had target recognition isolated from signal generation; these two reactions took place in separate chambers that were only connected through an electrode. This configuration allowed us to (i) independently optimize each reaction type (i.e., target recognition and signal generation), (ii) devise the electrode shape for maximal sensitivity, and (iii) lay out a compact array for higher throughput. To prove the concept, we developed an ECLipse prototype for multiplexed detection of inflammatory biomarkers [interleukin-3 (IL-3), IL-6, and procalcitonin (PCT)] (*14*). We designed a disposable cartridge with eight detection sites and integrated it with a portable detector. The system achieved superior sensitivity (>7000-fold better than ELISA) and detected multiple markers in parallel within an hour. In pilot clinical studies, we applied ECLipse to identify patients with sepsis and stratify their survival; we could further confirm distinct CK patterns in coronavirus disease 2019 (COVID-19) patients with severe acute respiratory syndrome coronavirus 2 (SARS-CoV-2) infection.

## RESULTS

### ECLipse assay

**Fig. 1.**
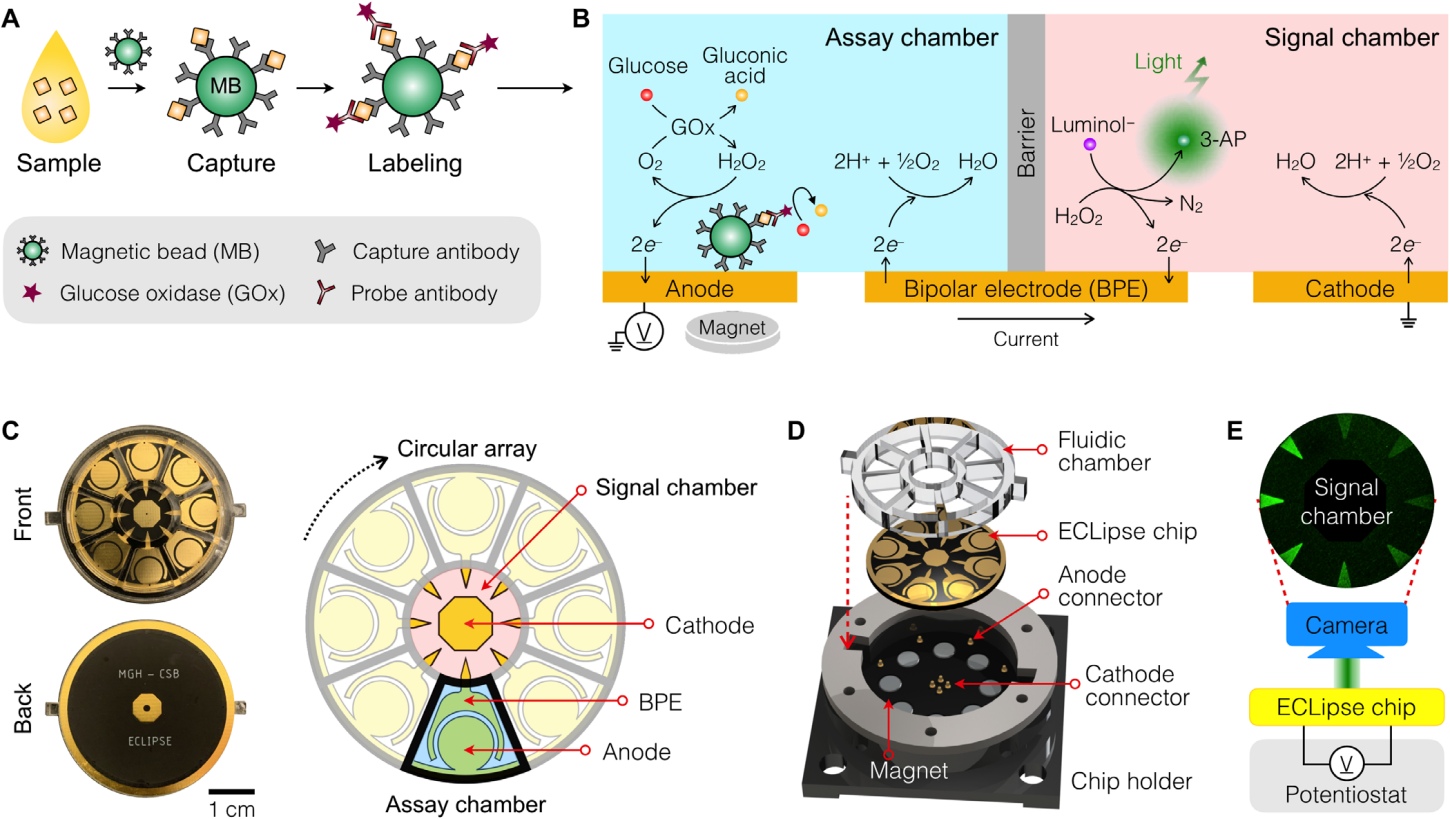
ECLipse. (**A**) In ECLipse, the biorecognition reaction is separated from signal generation. The biorecognition assay uses immunomagnetic beads to capture protein targets directly from clinical samples. The targets are further labeled with oxidizing enzymes [glucose oxidase (GOx)] through probe antibodies. (**B**) ECLipse schematic. Two isolated compartments, an assay and a signal chambers, are connected solely through a BPE. The assay chamber is loaded with enzyme-labeled beads that are magnetically enriched on the anode; the signal chamber contains ECL reagents (luminol, H_2_O_2_). With an electrical potential applied, the enzyme-labeled beads catalyze a redox reaction in the assay chamber, which leads to an ECL reaction in the signal chamber. The ECL intensity is proportional to target protein concentrations. The entire assay completes within 1 hour. (**C**) Schematic and images of an ECLipse chip. The circular chip had eight assay chambers in a radial array format and a shared signal chamber in the center. The chip was made on a printed circuit board (PCB). Electrodes were patterned on the front side and electrical contacts on the backside. (**D**) Exploded view of the system assembly. A fluidic structure dividing reaction chambers was attached to the ECLipse chip. The chip assembly was mounted on a device holder which had pogo pins as electrical contacts and magnets at anode locations. (**E**) Measurement setup. The ECLipse chip was connected to a single potentiostat, and the ECL signal from the signal chamber was imaged by a camera.

The assay starts with immunocapturing molecular targets with magnetic beads. Captured targets are further labeled with probe antibodies (Fig. 1A), and the bead-antibody complexes are introduced to the ECLipse chip for detection (see fig. S1 for the workflow). The assay completes within 1 hour, and native samples (e.g., plasma and urine) can be used without purification. The ECLipse chip has two electrolytic cells (Fig. 1B): an assay chamber with an anode and a signal chamber with a cathode. These chambers are physically isolated by a fluidic wall but electrically coupled through a common BPE (*15*–*17*). Applying an external voltage between the anode and the cathode triggers EC reactions in both chambers: oxidation at the anode (assay chamber) and reduction at the cathode (signal chamber). The BPE functions as a pseudo-ground that keeps the charge neutrality in both chambers: Reduction reaction occurs on the assay-side BPE, and oxidation on the signal side.

For biosensing, we paired an immunodetection with an ECL generation. In the assay chamber, we loaded glucose along with bead-antibody complexes that were labeled with oxidizing enzymes [glucose oxidase (GOx)]. These beads were magnetically concentrated onto the anode. In the signal chamber, we loaded a mixture of luminol and hydrogen peroxide (H_2_O_2_). The labeled GOx at the bead-antibody complex metabolizes glucose and oxygen to produce gluconic acid and H_2_O_2_. Under an electrical potential, H_2_O_2_ further underwent EC oxidation on the anode surface in the assay chamber, and the resulting faradaic current flowed through the BPE to set off luminol oxidation in the signal chamber (see table S1 for details). This arrangement would achieve high signal-to-background ratio; ECL was generated only when the electrical current was flown from the assay chamber. Using luminol as a luminophore also minimized the risk of side reactions (e.g., electrolysis of water) because it could be oxidized at low potential (<0.5 V) (*18*).

### ECLipse detection system

With the assay and the signal chambers separated, we could engineer electrodes optimal for each reaction type (Fig. 1C). In the assay chamber, the anode occupied the most surface area, a design feature that promotes direct contact between magnetic beads and the electrode, thereby lowering the charge transfer resistance. The BPE counterpart surrounded the anode to make a uniform coupling. In the signal chamber, we tapered the BPE toward the cathode, which enhanced the ECL intensity by concentrating electrons at the BPE tip. Moreover, the cathode could be shared among different BPEs because ECL reactions were localized. This advantage made it easy to scale up the ECLipse chip for higher throughput; a circular array of assay chambers could be laid out, with a common signal chamber at the center (Fig. 1C). The prototype chip had eight detection sites and was manufactured on a printed circuit board (PCB) (see Materials and Methods for details).

Figure 1D shows the overall chip assembly. We bonded a plastic fluidic structure on the ECLipse chip to define assay and signal chambers. The assembly was then mounted on a custom-made chip socket. Pogo pins in the socket made electrical contacts with the chip, and an embedded magnet array concentrated magnetic beads on top of each anode. For signal detection, we applied an electric potential between anodes and a common cathode and imaged the signal chamber (Fig. 1E). One potentiostat powered the entire array, requiring only two external electrical connections, and all eight measurements were carried out simultaneously.

### Electrode design for the ECLipse system

**Fig. 2.**
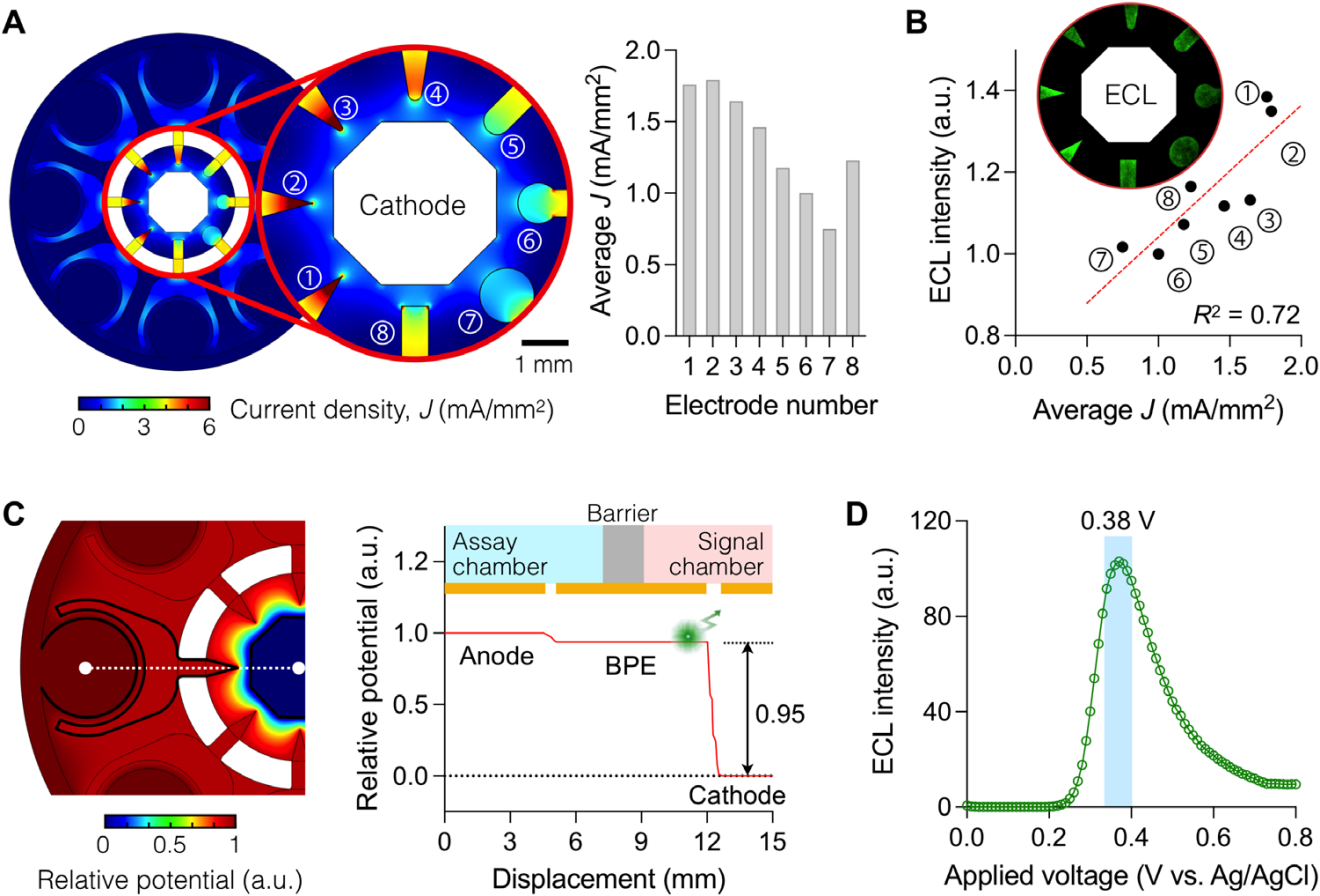
Electrical characteristics of the ECLipse chip. (**A**) Electrical current density (*J*) among different electrode designs. The curvature of electrodes was varied in the signal chamber (left). Tapered electrodes had higher *J* values than flat or round ones (right). Electrodes are numbered in the plots for identification. (**B**) ECL intensity was measured (inset) from a test chip that had the same design shown in (A). The light intensity is linearly correlated (*R*^2^ = 0.72) with the estimated current density. Electrode 1, which generated the highest ECL, was chosen for the ECLipse chip. The applied external potential was 0.4 V between the anode and the cathode. (**C**) Electrical potential was calculated for the ECLipse chip with the final electrode design (left), and the potential profile was plotted along the white dashed line (right). Most voltages dropped (95%) inside the signal chamber. This profile benefitted the ECLipse assay because luminol oxidation in the signal chamber required higher electrical potential than H_2_O_2_ oxidation in the assay chamber. (**D**) ECL intensity from luminol peaked at the potential difference of 0.38 V in the signal chamber. Following this result, the operation potential was set to 0.4 V (= 0.38 V/0.95) between the anode and the cathode. a.u., arbitrary units.

We first determined the BPE shape to maximize the ECL signal. We reasoned that increasing the electrical current density (*J*) would generate a high ECL signal. The ECL intensity (*p*) scales with the photon flux, *p* ~ (1/*A*)·(*dn_p_*/*dt*), where *n_p_* is the number of photons created and *A* is the surface area of an electrode. The photon number *n_p_* is proportional to the number of electrons (*n_e_*) from an electrode (*n_p_* ~ *n_e_*), which leads to *p* ~ (1/*A*)·(*dn_e_*/*dt*) ~ *I* / *A ~ J*, where *I* is the electrical current (*19*). To test this hypothesis, we prepared a test chip containing BPEs of various tip shapes (fig. S2). The current density *J* was estimated from numerical simulations (Fig. 2A), which showed higher *J* in electrodes with sharper tips. The ECL intensity measured from these BPEs followed the same trend (Fig. 2B, inset), showing a linear correlation with the estimated *J* (Fig. 2B). From these results, we chose the triangular-tipped BPE (fig. S3).

We further analyzed the electrical potential distribution inside the ECLipse chip (Fig. 2C). Across the electrodes, the most voltage dropped (95%) between the BPE and the cathode. This profile was preferable because luminol oxidation in the signal chamber would require a higher voltage (>0.30 V) (*20*, *21*) than H_2_O_2_ oxidation in the assay chamber (>0.04 V; fig. S4). We further observed that the luminol signal reached its maximum with the potential difference of 0.38 V between the BPE and the cathode (Fig. 2D). The overall operation voltage was thus set to 0.4 V (=0.38 V/0.95).

### Optimizing the ECLipse assay

**Fig. 3.**
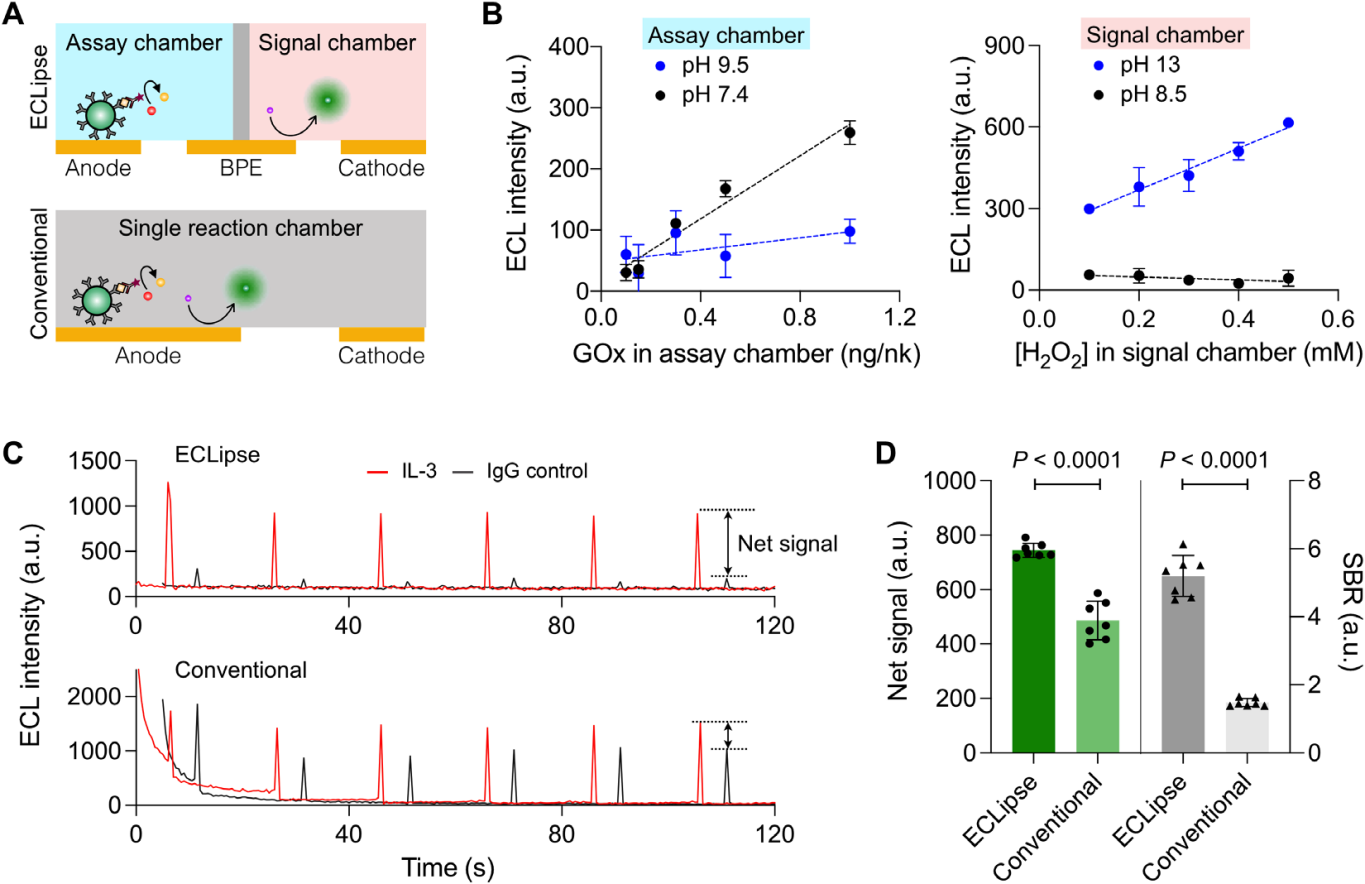
Optimizing the ECLipse assay. (**A**) ECLipse used two separate reaction settings that were optimal for glucose oxidation and ECL generation, respectively. In the conventional ECL setup, both reactions took place in a single chamber, which limited assay optimization. (**B**) Optimal buffer conditions for the ECLipse assay. Buffers of different pH were used in the assay chamber [phosphate-buffered saline (PBS), pH 7.4; carbonate buffer, pH 9.5) and in the signal chamber (tris-HCl, pH 8.5; KOH, pH 13). In the assay chamber (left), glucose oxidation was efficient under a neutral buffer (pH 7.4) condition. In the signal chamber (right), a strong basic buffer led to a high ECL signal, as the solubility of luminol improved. Data are displayed as means ± SE from duplicate measurements. (**C**) Comparison of signal intensity between ECLipse and conventional ECL assays. Targeted samples (IL-3) were prepared using magnetic beads conjugated with capture antibody; control samples were prepared using magnetic beads with immunoglobulin G (IgG) antibody. ECL signal was generated when potential pulses were applied (pulse width, 0.1 s). ECLipse had lower background than the conventional ECL assay. (**D**) ECLipse showed net signal (targeted − control) and signal-to-background ratio (SBR; targeted/control) that were significantly higher than those by conventional ECL measurements (unpaired two-sided *t* test, *P* < 0.0001 for both metrics).

We next optimized the ECLipse assay protocol. Neutral pH was preferred for the effective enzymatic activity of GOx, whereas basic pH (>10) was required to increase luminol’s solubility (*21*). With their one-chamber design, conventional ECL systems cannot meet these requirements and typically operate at a weak basic pH (8.5). ECLipse, in contrast, allowed us to optimize each reaction type to achieve maximal sensitivity (Fig. 3A). For example, we could enhance the activity of GOx (i.e., H_2_O_2_ production) by keeping a physiological pH (7.4) in the assay chamber (Fig. 3B, left). In the signal chamber, both the ECL signal and its resolving power improved under strongly basic conditions (Fig. 3B, right).

The optimized ECLipse showed a superior analytical performance (Fig. 3C). We prepared on-target samples wherein IL-3 was immunocaptured and labeled with probe antibodies. Control samples followed the same labeling processes but used magnetic beads conjugated with immunoglobulin G (IgG) antibodies. ECL reaction was triggered by pulsing an electrical potential. ECLipse generated more consistent ECL peaks than the conventional system: The coefficients of variation were 1.7% (ECLipse) and 7.3% (conventional ECL). ECLipse also had negligible background when no electrical potential was applied (fig. S5). The conventional assay, however, showed higher background. A likely cause was the side reaction between glucose and luminol; we observed decaying chemiluminescence and ECL in the mixture of glucose and luminol (fig. S6). ECLipse was free from this side reaction because glucose and luminol were physically separated. We used a net signal difference between on-target and IgG samples to account for signals from nonspecific target capture. Overall, ECLipse reported a higher (150%) net signal (=IL-3 sample – IgG control) and higher (370%) signal-to-background ratios (=net signal/IgG control) than the conventional ECL system (Fig. 3D).

### Assay characterization

Applying the optimized assay condition, we characterized the ECLipse analytical performance. Immunomagnetic beads specific to IL-3, IL-6, or PCT were prepared; control beads were conjugated with IgG antibodies (see Materials and Methods for details). We made test samples by spiking varying amounts of target proteins into human plasma. These samples were mixed with immunomagnetic beads and then labeled with GOx via probe antibodies. Enzyme-labeled beads and glucose were then loaded in the assay chamber (pH 7.4); luminol and a coreactant (H_2_O_2_) in the signal chamber (pH 13). We initiated the ECL reaction by applying the electrical potential (0.4 V) between the anode and the cathode. We used the signal difference between targeted and control samples as an analytical measure, thereby accounting for nonspecific protein binding.

**Fig. 4.**
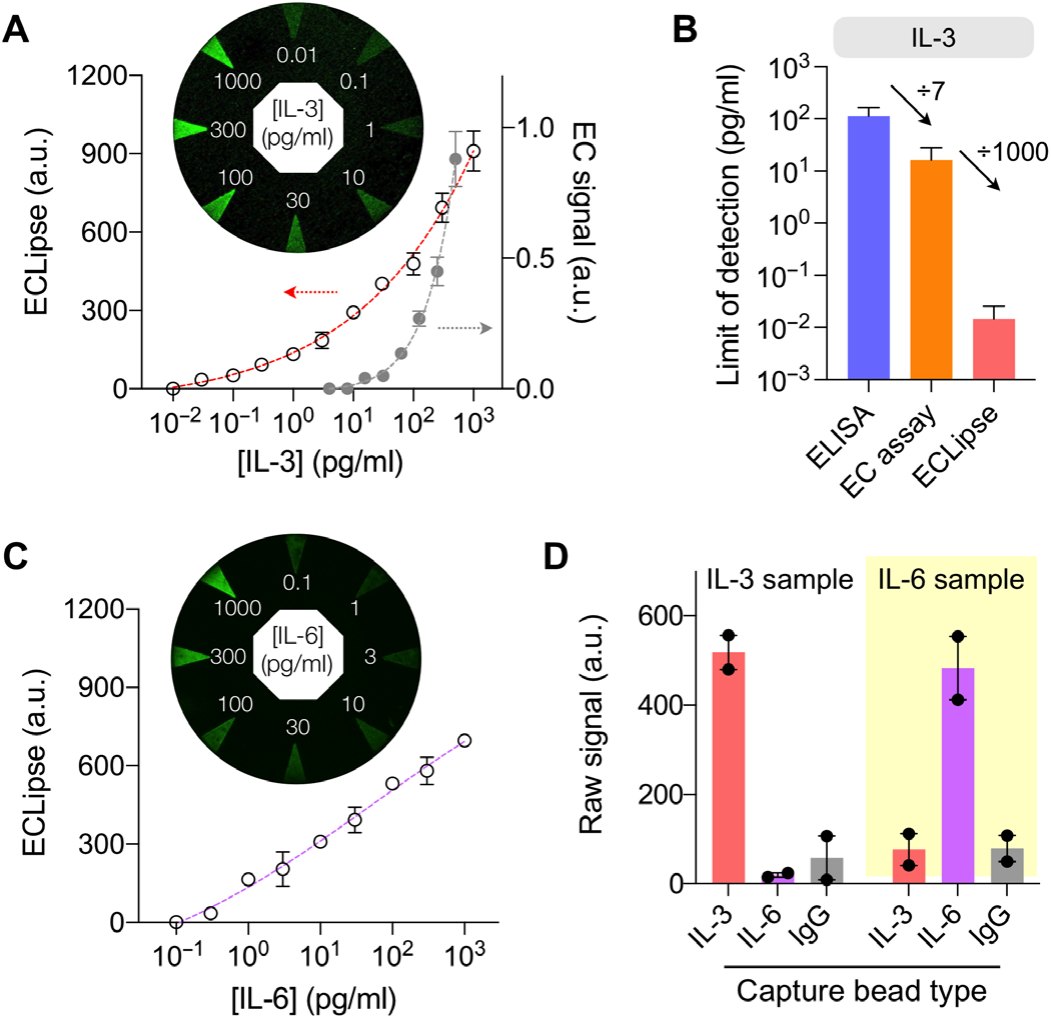
Characterization of the ECLipse assay. (**A**) ECLipse and EC assays were used to quantify IL-3 spiked in human plasma samples. Background signal was subtracted that arose from innate IL-3 in plasma. The ECLipse assay had a lower limit of detection (LOD) and a wider dynamic range (DR) than the EC assay. Inset: ECL image of an ECLipse chip assessing samples with varying IL-3 concentrations. (**B**) LODs were compared among different assay modalities. ECLipse (LOD, 15 fg/ml) was about 1000-fold more sensitive than EC assay (LOD, 16 pg/ml) and about 7000-fold more than ELISA (LOD, 113 pg/ml). Data are shown as means ± SD from triplicate measurements. (**C**) ECLipse assay of plasma samples spiked with varying amounts of IL-6. Inset: ECL image of an ECLipse chip. (**D**) Assay selectivity. Samples containing IL-3 (100 pg/ml) or IL-6 (100 pg/ml) were processed for IL-3, IL-6, and control assays. On-target samples produced high ECL signals, whereas off-target samples had signal levels close to or lower than those of IgG controls.

Figure 4A shows the titration results with IL-3 samples. ECL intensities on electrodes were visibly dependent on IL-3 concentrations (Fig. 4A, inset). The IL-3 titration curve, generated from the mean ECL intensity per electrode, established the limit of detection (LOD) of 15 ± 11 fg/ml (from technical replicates; *n* = 3) and the dynamic range (DR) spanning five orders of magnitude. On both metrics (i.e., LOD and DR), ECLipse surpassed a conventional ELISA (fig. S7) and an EC assay (Fig. 4A). ECLipse’s LOD was markedly lower, with the improvement factors of 7000 over ELISA and 1000 over the EC assay (Fig. 4B), and ECLipse DR (10^5^) was wider than those of both assays (DR ~ 10^2^, ELISA; DR ~ 10^3^, EC assay). Titration experiments with IL-6 (Fig. 4C) and PCT (fig. S8) further confirmed ECLipse’s high sensitivity, with ECLipse having lower LODs (IL-6: 123 ± 7 fg/ml, *n* = 3; PCT: 230 ± 38 fg/ml, *n* = 3) than ELISA (IL-6: 19.6 pg/ml; PCT: 28.7 pg/ml). ECLipse assays also were highly specific to their intended targets (Fig. 4D). With antibodies screened for minimal cross-reactivity, we could maintain off-target signals at the level of IgG controls (see table S2 for the list of antibodies).

ECLipse demonstrated good analytical reproducibility (fig. S9). We could accurately quantify target concentrations by looking up measured raw data in the titration curve. Repeatedly measuring the same sample yielded consistent results (i.e., high precision) in intra- and interassay validations. Using immunomagnetic beads was also found to minimize the matrix effect. Once the target was captured, magnetic beads were transferred and resuspended in a buffer for subsequent reactions. When we measured two types of test samples, target proteins spiked in either phosphate-buffered saline (PBS) or human plasma, we observed no notable signal difference between the two matrices (fig. S10).

### ECLipse assay for sepsis detection

As a pilot clinical study, we applied ECLipse for sepsis diagnosis. Sepsis is a frequently fatal condition characterized by an uncontrolled and harmful host reaction to infection (*1*, *22*). Accurate early diagnosis is critical to improving patient survival, considering that sepsis can rapidly progress to septic shock and death within a few hours of disease onset (*23*). We chose three proteins as candidate biomarkers, IL-3, IL-6, and PCT, that are known to be elevated in sepsis (*1*, *24*–*26*). Plasma samples (*n* = 35) from patients with sepsis were collected within 3 days of suspected septic onset; control samples (*n* = 35) were collected from healthy donors (see table S3 for clinical information). For a given, undiluted plasma sample, we used a single ECLipse chip to detect multiple targets (i.e., IL-3, IL-6, PCT, and IgG control) and obtained the net signal difference between on-target and IgG samples. Target concentrations were then estimated based on pregenerated calibration curves (Fig. 4 and fig. S8).

**Fig. 5.**
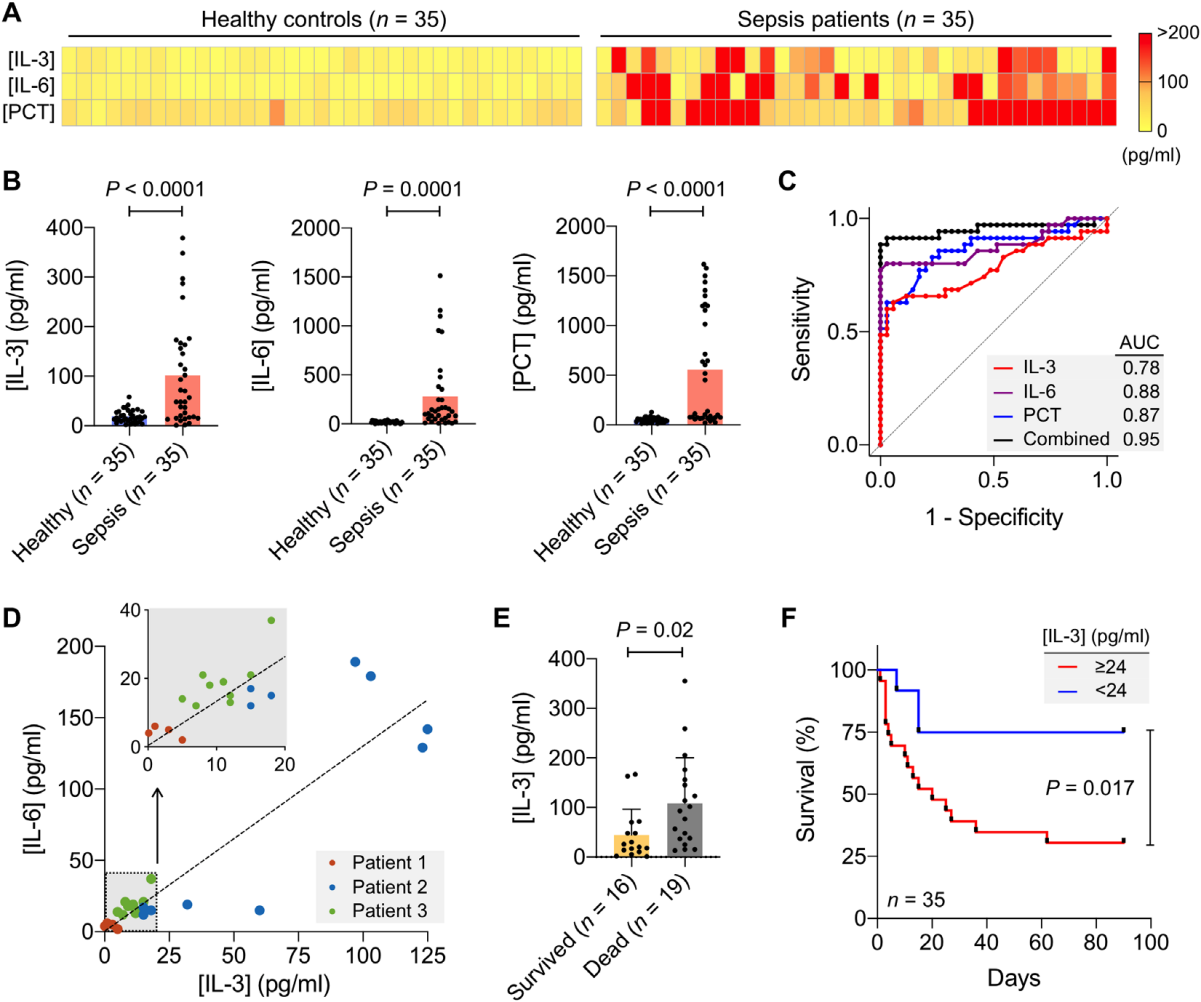
ECLipse for sepsis diagnosis. (**A**) ECLipse was used to quantity three sepsis markers (IL-3, IL-6, and PCT) in clinical plasma samples. The heatmap contrasts the marker distribution between healthy controls (*n* = 35) and patients with sepsis (*n* = 35). (**B**) IL-3, IL-6, and PCT concentrations in plasma were significantly higher (unpaired two-sided *t* test) in patients with sepsis than in nonseptic controls. (**C**) Receiver operating characteristic (ROC) curves were constructed to compare the diagnostic ability of IL-3, IL-6, and PCT. Combining all three markers increased the area under the curve (AUC) to 0.95. (**D**) Plasma samples of three patients with sepsis were monitored over the course of the disease. We observed a positive correlation (Pearson’s *r* = 0.91) between IL-3 and IL-6 concentrations. Inset: Zoomed-in view at low IL-3 and IL-6 concentrations. (**E**) IL-3 concentrations of patients with sepsis (*n* = 35) measured on the first day admitted to the hospital. When grouped according to the clinical outcome, IL-3 levels were significantly higher (unpaired two-sided *t* test) in patients with sepsis who later died of the disease. (**F**) Survival analysis. Patients with sepsis from (E) were stratified by the [IL-3] threshold (24 pg/ml) that was previously determined. The Kaplan-Meier estimator for the patient survivor showed that the cohort with high [IL-3] was associated with poor prognostics (*P* = 0.017, two-sided log-rank test).

Figure 5A shows marker profiles among sepsis and normal plasma samples. The average concentration of each protein was significantly higher in patients with sepsis than in normal controls (*P* < 0.0001, IL-3; *P* = 0.0001, IL-6; *P* < 0.0001, PCT; unpaired two-sided *t* test), which supported the potential use of these markers for sepsis detection (Fig. 5B). To assess the diagnostic power of each marker, we further generated an operating characteristic curve (Fig. 5C). Single markers had the area under the curve (AUC) values of 0.78 (IL-3), 0.88 (IL-6), and 0.87 (PCT). Multiplexed detection was key to robust diagnostics: With a three-marker combination, the AUC increased to 0.95 (see Table 1 for the summary of diagnostic statistics).

**Table 1.** Sepsis diagnosis statistics by ECLipse.

**Markers**			**Cohort (*n* = 70)**		
		**AUC**	**Sensitivity (%)**	**Specificity (%)**	**Accuracy (%)**
Single markers	IL-3	0.78	63 (45–79)*	94 (81–99)	79 (67–87)
	IL-6	0.88	80 (63–92)	97 (85–100)	89 (79–95)
	PCT	0.87	80 (63–92)	80 (63–92)	80 (69–89)
Marker combinations (weighted)	IL-3 + IL-6	0.93	89 (73–79)	97 (85–100)	93 (84–98)
	IL-3 + PCT	0.88	74 (57–88)	94 (81–99)	84 (74–92)
	IL-6 + PCT	0.94	83 (66–93)	100 (90–100)	91 (82–97)
	IL-3 + IL-6 + PCT	0.95	89 (73–97)	100 (90–100)	94 86–98)

*The numbers in parentheses indicate 95% confidence intervals.

We further examined IL-3 as a predictor of septic shock and death. Recent studies have shown that IL-3 plays an essential role during early sepsis: IL-3 operates upstream of key CKs [e.g., IL-6, IL-1β, and tumor necrosis factor–α (TNF-α)], and high IL-3 levels increase the risk of CK storms (*1*, *25*). To ascertain this link, we longitudinally monitored IL-3 and IL-6 in patients with sepsis (fig. S11). IL-3 and IL-6 concentrations showed an excellent positive correlation (Pearson’s *r* = 0.91; Fig. 5D), which could be attributed to the downstream effect of IL-3 on IL-6 production. IL-3 levels at sepsis onset were significantly higher (*P* = 0.02, unpaired *t* test) in patients who eventually succumbed to death (Fig. 5E), whereas IL-6 levels at sepsis onset showed no significant difference (*P* = 0.375, unpaired *t* test) between nonsurvived and survived patients (fig. S12). Survival analysis further confirmed a significant association of IL-3 with patient’s death (Fig. 5F). We stratified patients with sepsis into two groups, IL-3 high versus low, according to the [IL-3] cutoff (=24 pg/ml) from a previous survival study (*25*). Patients in a high IL-3 group showed about a fourfold greater hazard of death than those with low levels (*P* = 0.017 for log-rank test; hazard ratio, 3.9).

### ECLipse analysis of patients with COVID-19

**Fig. 6.**
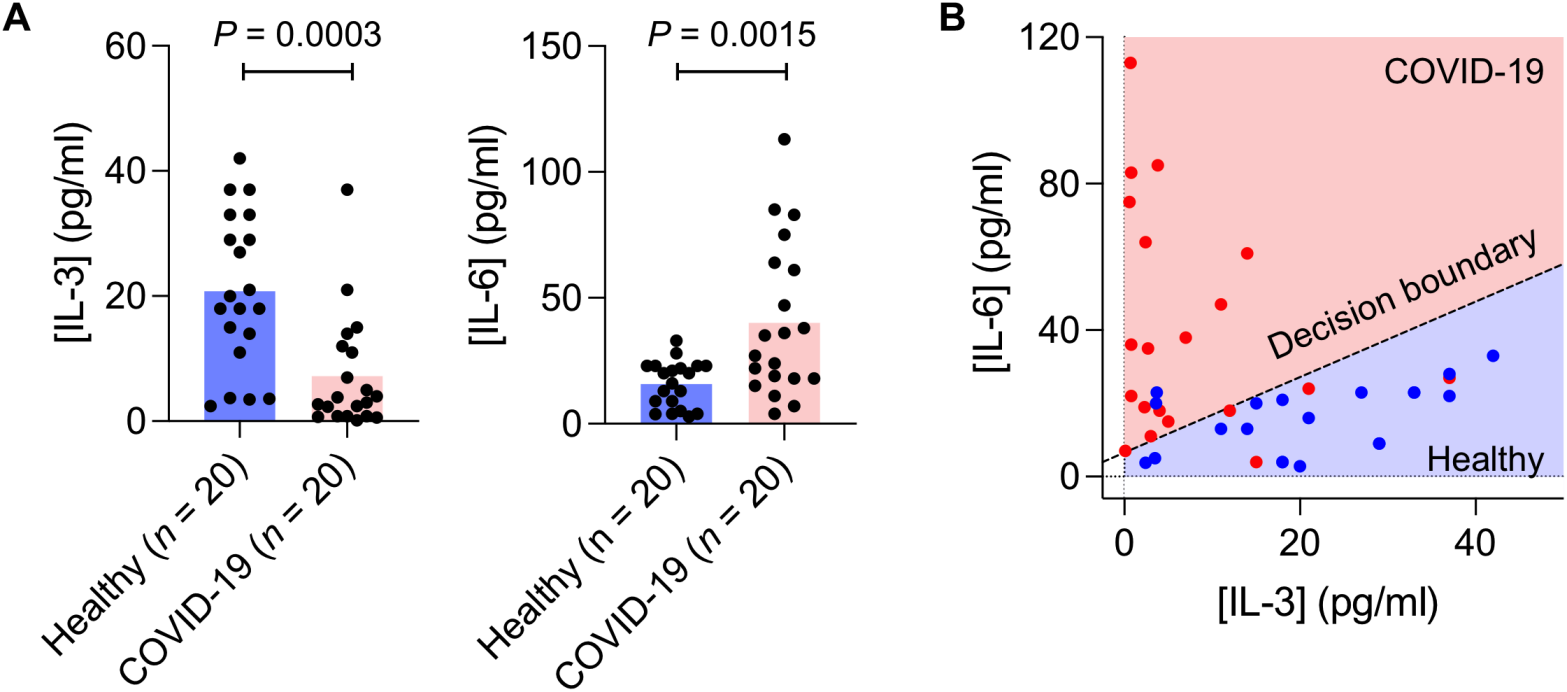
ECLipse assay of COVID-19 patient samples. (**A**) Plasma samples from patients with confirmed COVID-19 (*n* = 20) were analyzed for IL-3 and IL-6. Control samples (*n* = 20) were from healthy donors. IL-3 and IL-6 concentrations showed an opposite trend: IL-3 levels were significantly lower (*P* = 0.0003, unpaired two-sided *t* test), and IL-6 levels were significantly higher (*P* = 0.0015, unpaired two-sided *t-*test) in patients with COVID-19 than in noninfected controls. (**B**) A logistic regression model separated patients with COVID-19 from controls, using IL-3 and IL-6 levels as predictors. The classification accuracy was 85%. The dashed line indicates the decision boundary.

We lastly applied ECLipse to analyze IL-3 and IL-6 in plasma samples of patients with COVID-19 (table S3). These two markers are increasingly recognized as potential predictors of severe COVID-19 (*27*, *28*). Our recent study showed that IL-3 promotes the recruitment of antiviral plasmacytoid dendritic cells into the airway, which connected low IL-3 levels with increased severity during SARS-CoV-2 infections (*27*). For IL-6, the association is reversed: Many clinical studies have found high IL-6 concentrations in severely ill patients with COVID-19 (*28*–*30*). ECLipse tests supported such an opposing trend. We analyzed plasma samples from patients with COVID-19 (*n* = 20) who were hospitalized for treatment. Patients with COVID-19 showed lower IL-3 and higher IL-6 concentrations in plasma than noninfected controls (Fig. 6A). Using IL-3 and IL-6 as predictor variables, we constructed a logistic regression model (Fig. 6B), which achieved a classification accuracy of 85%.

## DISCUSSION

ECL provides a robust mechanism for biosensing. Its analytical signal is free from the optical background and generated through a convenient electrical control (*31*). The closed BPE systems offer additional controllability by physically separating the anode and the cathode reactions. In ECLipse, we exploited this unique mechanism to further improve BPE’s analytical capabilities. First, we independently optimized the reaction condition for ECL generation and target recognition (Fig. 3B), improving the assay sensitivity while minimizing cross-reactions. Second, we engineered the electrode shape for maximal efficiency. On the target recognition side, electrodes had a large surface area to enhance the coupling with electrolytes; and on the ECL generation side, the electrode had a sharp tip to concentrate electrons and thereby increase light intensity (Fig. 2B). Third, we used one cathode shared by many BPEs (Fig. 2C), which was possible because the ECL reaction was localized on the BPE tip. This finding allowed us to lay out a radial array to increase the assay throughput while requiring only two electrical connections. In our proof-of-concept study, we built a system to detect eight targets simultaneously. The system achieved higher signal-to-background contrasts than the conventional ECL method and surpassed ELISA and the EC assay in sensitivity.

Analyzing patient samples demonstrated ECLipse’s potential for clinical uses. Besides being highly sensitive, the ECLipse assay had a wide DR to seamlessly detect multiple markers from a single parent sample. These capacities were essential when ECLipse was applied to identify sepsis in clinical plasma samples: Measuring multiple host factors (i.e., IL-3, IL-6, and PCT) led to high diagnostic accuracy (AUC = 0.93). We also reaffirmed the emerging recognition of IL-3 as a predictor of septic shock and death. In plasma samples collected at sepsis onset, ECLipse assays reported higher IL-3 concentrations in patients who eventually died of sepsis. Moreover, the IL-3 cutoff (=24 pg/ml), established from a previous study (*25*), remained effective in classifying patients into high or low death risk groups. These outcomes, combined with ECLipse’s speed (<1 hour for assay) and affordability ($1.5 USD for a chip), point to the possibility of sepsis diagnosis and treatment decision at point of care—an urgent technical need in sepsis care (*32*).

Measuring IL-3 and IL-6 in patients with COVID-19 produced intriguing results. The average IL-6 concentration was higher in patients with COVID-19 than in normal controls, whereas IL-3 showed the opposite behavior. Notably, IL-3 in patients with COVID-19 was difficult to quantify by ELISA because IL-3 concentration in this cohort (7 pg/ml ~ 0.4 pM, on average) was close to or below ELISA’s quantification limit. ECLipse, in contrast, could reliably resolve low IL-3 levels, revealing robust differences in IL-3 concentration between patients with COVID-19 and normal controls. These observations may help strategize proactive COVID-19 management: administering IL-6 receptor antagonists to high IL-6 patients to mitigate severe inflammatory conditions or recombinant IL-3 to patients with low IL-3 to improve antiviral defense (*27*–*29*, *33*). In these clinical scenarios, ECLipse can identify patients for optimal treatment and monitor its efficacy afterward.

We envision improving several aspects of the current study. First, we could redesign the ECLipse chip for higher throughput. This task can be readily achieved by increasing the number of circular arrays in the ECLipse chip. The existing detector can be used without modification because this new chip would need only two-electrical connections. However, we may proceed with caution. As more BPEs are laid out, their interdistance would decrease on the cathode side, which will increase the risk of cross-talk. Second, we need to automate the sample processing, ideally as a built-in function of the assay system (sample-in and answer-out). This integration will ensure reliable tests by minimizing hands-on times and promote ECLipse’s adoption in busy clinical settings (e.g., emergency department). We expect that ECLipse’s operation can be readily automated. ECLipse uses magnetic beads as a substrate, which can be easily controlled by external magnets (i.e., no need for centrifugation). A robotic manipulator thus can wash and transport magnetic beads between different reagents (*25*, *34*). Third, we should expand the study to obtain rigorous assay statistics. The assay robustness (e.g., accuracy and precision) should be further validated among different ECLipse chips and detection systems. In addition, increasing the patient cohort would facilitate validating the current marker sets and refining their cutoffs. We could consider including other host response markers (e.g., TNF-α, chemokine ligand 12, and interferon-γ) (*14*) and endotoxins to improve diagnostic accuracy. With these advances, ECLipse will be a powerful clinical tool to enable early infection diagnosis and improve treatment outcomes.

## MATERIALS AND METHODS

### Design and fabrication of ECLipse chips

The ECLipse chip consisted of a PCB and a fluidic chamber. The PCB had double conducting layers; a circular array of electrodes was patterned on the top side and electrical contacts on the bottom. Electrodes were plated with gold (thickness, 76 μm) through the electroless plating process (nickel/immersion gold). The fluidic chamber was made in plastic; we used a laser cutter (Fusion Edge 24, Epilog Laser) to lay out the chamber on an acrylic sheet laminated with an adhesive. The prepared chamber (thickness, 4.5 mm) was then attached to the PCB.

### Numerical simulation of electric potential and current

We simulated the electrical property of the ECLipse chip using a commercial package (COMSOL Multiphysics, Microfluidics Module). All electrodes were assumed to be made of gold (conductivity, 6.0 × 10^7^ S/m), and the shape of BPEs was varied to test its effect on the ECL signal. The assay and the signal chambers were assigned with electrical conductivity values of electrolytes used in experiments: 0.35 S/m (phosphate buffer, assay chamber); 20 S/m (0.1 M KOH, signal chamber). We used the electric current stationary solver to obtain the current density and the electrostatic solver to simulate the electrical potential inside the ECLipse chip.

### ECLipse measurement setup

We constructed a socket-type holder to mount an ECLipse chip and connect it to a potentiostat (Model 301A, Graphenide Technology). The holder had an array of permanent NdFeB magnets (diameter, 6.35 mm) to concentrate magnetic beads onto anodes and gold-plated pogo pins (P70-2010045R, Mouser Electronics) to make electrical contacts with the ECLipse chip. The holder body was made in plastic via three-dimensional printing (Form2, Formlabs). For ECL detection, we used a charge-coupled device (CCD) camera (Falcon II, Raptor Photonics). We placed a focusing zoom lens (FB0745, Guilin FT-OPTO) and a band-pass filter (360 to 580 nm) in front of the camera and imaged the entire signal chamber.

### Preparation of immunomagnetic beads

We used magnetic beads (diameter, 2.7 μm) coated with an epoxy group (Dynabeads M-270 Epoxy, Invitrogen). Beads (1-mg solid weight) were added to a sodium phosphate solution (200 μl, 0.1 M), and the mixture was placed on a 360-rotating mixer (HulaMixer, Thermo Fisher Scientific) for 10 min at 20°C. We then collected washed beads using a permanent magnet and resuspended them in a sodium phosphate solution (100 μl, 0.1 M). For antibody conjugation, we added 20 μg of antibodies (1 mg/ml) into a 100-μl bead solution along with ammonium sulfate (100 μl, 3 M) to increase the ionic strength of the solution. The mixture was allowed to react (8 hours at 4°C) on the rotating mixer. We then triple-washed beads in PBS (magnetic washing) and resuspended them in PBS (50 μl) containing 1% bovine serum albumin (BSA). The final bead concentration was ~10^9^ bead/ml. Antibody information is in table S2.

### ECLipse assay

To capture protein targets, we added immunomagnetic beads (10^9^ bead/ml, 2 μl) into a plasma sample (100 μl) and incubated the mixture on the rotating mixer (20°C for 30 min). The beads were magnetically collected, washed twice in PBS containing 0.05% Tween 20 (PBS-T), and mixed with a solution of biotinylated probe antibodies (5 μg/ml, 30 μl in 0.1% BSA). After a brief incubation (10 min at 20°C) period, beads were washed in PBS-T and labeled with GOx; we used a solution (30 μl) containing GOx (10 μl/ml in 0.1% BSA) conjugated with streptavidin. The mixture was incubated for 5 min at 20°C. Last, labeled beads were collected, washed in PBS, and resuspended in PBS (30 μl). For spiked-in experiments with human plasma, we adjusted the signal for innate target proteins. Specifically, we analyzed neat samples (i.e., nonspiked-ins) and subtracted their signals from those of spiked-in samples.

### ECLipse signal detection

To generate an ECL signal, we injected the labeled beads (7 μl) and a glucose solution (0.2 mM, 200 μl in phosphate buffer) into the assay chamber (pH 7.5). The signal chamber (pH 13) was loaded with luminol (10 mM, 200 μl in 0.1 M KOH) and hydrogen peroxide (4 mM, 5 μl). We started the ECL reaction by applying a constant electrical potential (0.4 V) between anodes and the cathode in the ECLipse chip and imaged the signal chamber using the CCD camera. The exposure time was set to 60 s. From the acquired images, we extracted the areal average of ECL intensities (ImageJ). Using a sensitive camera will shorten the acquisition time. In the photon-noise limited case, the signal-to-noise ratio (SNR) of a CCD camera scales as SNR ~ (*P* · *Q_e_* · *t*)^0.5^, where *P* is the photon flux, *Q_e_* is the quantum efficiency, and *t* is the exposure time. A camera with higher *Q_e_* thus can achieve a target SNR in a shorter exposure time.

### Conventional (signal chamber) ECL assay

Magnetic beads labeled with GOx were prepared as described above. We mixed the beads (7 μl in PBS) with a glucose solution (0.2 mM, 38 μl) in 10 mM tris-HCl buffer (pH 8.5) and then added into the mixture a luminol solution (25 μM, 5 μl) in 10 mM tris-HCl buffer (pH 8.5). After 2-min incubation (20°C) to generate hydrogen peroxide via enzymatic reaction, we loaded the mixture on a screen-printed carbon electrode (SPE 110, Metrohm DropSens). For ECL generation, we next applied repeated pulses (0.38 V, 0.1-s duration) every 20 s and measured the signal by a photomultiplier tube (H10682, Hamamatsu). ECL signals from the first five pulses were averaged.

### Enzyme-linked immunosorbent assay

Antibodies (4 μg/ml; see table S1 for antibody information) were loaded (50 μl per well) in a 96-well plate (Nunc MaxiSorp flat-bottom, Thermo Fisher Scientific) for adsorption (12 hours at 4°C). After washing twice with PBS (200 μl) containing 0.1% BSA, we treated the plate with 1% BSA (200 μl per well) for 2 hours at 20°C, washed it twice, and then loaded it with plasma samples (50 μl per well) for target capture (2 hours, 20°C). After washing twice as described above, we added biotinylated detection antibodies (500 ng/ml, 50 μl per well) for target labeling (1 hour at 20°C). After washing out excess antibodies, we added horseradish peroxidase–conjugated streptavidin (1:20,000 diluted in 0.1% BSA; #405210, BioLegend) to each well (50 μl), waited for 20 min (20°C), and washed the plate three times. Last, we added 3,3′,5,5′-tetramethylbenzidine (100 μl, BioLegend) to each well and let the mixture react (30 min at 20°C). We stopped the reaction by adding a stop solution (50 μl) and read optical absorbance at 450 nm (Tecan).

### Statistical analyses

We used Prism version 9.3 (GraphPad Software Inc.) or R version 4.0 for all statistical analyses. We tested group differences using an unpaired *t* test. All tests were two-sided, and *P* < 0.05 was considered statistically significant. For the receiver operating characteristic (ROC) analyses, we used IL-3, IL-6, and PCT concentrations as predictors. We also considered the linear combination of these markers as a predictor. Optimal weights were determined via logistic regression. We then constructed ROC curves and determined level cutoff values that maximized the sum of sensitivity and specificity. Standard formulas were used to define sensitivity (true-positive rate), specificity (true-negative rate), and accuracy [(true positive + true negative)/(positive + negative)]. We calculated exact 95% confidence intervals for the sensitivity, specificity, and accuracy based on binomial probabilities (Clopper-Pearson). Analyses were performed using the pROC package (version 1.18.0) and the Hmisc package (version 4.7-0) in R. For survival analysis, we used the Kaplan-Meier method to calculate the survival functions stratified by IL-3 levels and the log-rank test to compare times to death between low and high IL-3 groups.

### Collection of clinical samples

We prospectively acquired clinical samples through the SEPICER trial. The SEPICER trial was first approved by the local ethics committee on 1 February 2016 (UKER 10_16 B) and modified on 28 April 2020 (UKER 174_20 B) and was conducted in the surgical intensive care unit of University Hospital of Erlangen, Germany. We enrolled patients who had an onset of sepsis syndrome ≤72 hours. Patients with sepsis and septic shock were classified according to the criteria of the International Sepsis Definitions Conference (*35*). Additional sepsis samples were collected at University Hospital of Giessen; the study was approved by the local ethics committee (Justus Liebig University of Giessen, trial code: 86/18). We determined this sample size to achieve sufficient power in evaluating ECLipse’s diagnostic statistics. We used the AUC as an index of accuracy. Our null hypothesis was that the ECLipse test has a similar AUC of ELISA (AUC = 0.71), whereas the alternative hypothesis was that the ECLipse performs significantly better (AUC ≥ 0.9). The sample size we used (35 sepsis versus 35 nonsepsis) would achieve 90% power to detect this AUC difference of 0.19 at a significance level of 5%. For COVID-19, we enrolled patients who were positive for SARS-CoV-2 polymerase chain reaction from oral swabs, oral fluid, or bronchoalveolar lavage fluid. Written informed consents were obtained from the study patients or their legal designees. The initial blood draw was also performed within this period. The management of patients with sepsis in the intensive care unit included early goal-directed therapy, elimination of the septic focus, and broad-spectrum antibiotics. Blood samples were collected, plasma was isolated for further analysis, and results were correlated to the clinical outcome.

## Notes

View/request a protocol for this paper from *Bio-protocol*.
