# Supplementary material for: Electrochemiluminescence in paired signal electrode (ECLipse) enables modular and scalable biosensing
Source: Sci Adv. 2022 Sep 21;8(38):eabq4022. doi: 10.1126/sciadv.abq4022 (PMC9491722; doi:10.1126/sciadv.abq4022)
Supplement: Supplementary file 1 — Figs. S1 to S12 Tables S1 to S3 [file sciadv.abq4022_sm.pdf]

Science Advances
AAAS

Supplementary Materials for

Electrochemiluminescence in paired signal electrode (ECLipse) enables modular and scalable biosensing

Young Kwan Cho *et al.*

Corresponding author: Georg F. Weber, georg.weber@uk-erlangen.de; Ik-Soo Shin, extant@ssu.ac.kr; Hakho Lee, hlee@mgh.harvard.edu

Sci. Adv. **8**, eabq4022 (2022)
DOI: 10.1126/sciadv.abq4022

This PDF file includes:

Figs. S1 to S12
Tables S1 to S3

Table S1. Chemical reactions in the ECLipse chip. In the assay chamber, an enzymatic reaction of oxidase (GOx) oxidized glucose to generate hydrogen peroxide (H_2O_2), which was subsequently oxidized on the anode. On the BPE portion, oxygen was reduced. In the signal chamber, luminol and H_2O_2 were oxidized on the BPE portion and generated luminescent light.

Assay chamber	Anode	Glucose + O_2 → Gluconic acid + H_2O_2 (GOx, Enzymatic reaction)<br>H_2O_2 → O_2 + $2H^+$ + $2e^-$
	BPE cathode	$1/2O_2$ + $2H^+$ + $2e^-$ → H_2O
Signal chamber	BPE anode	Luminol + H_2O_2 → O_2 + $2H^+$ + N_2 + $2e^-$ + 3-AP*<br>3-AP* → 3-AP + hv
	Cathode reaction	$1/2O_2$ + $2H^+$ + $2e^-$ → H_2O

Table S2. List of antibodies, proteins, and an enzyme used in this study.

Name	Cat #	Vender
Human IL3 antibody	MAB603R-01M	R&D system
Human IL3 biotinylated antibody	BAF203	R&D system
Human IL6 antibody	14-7069-85	Invitrogen
Human IL6 biotinylated antibody	AHC0469	Invitrogen
Human PCT antibody	MA1-20888	Invitrogen
Human PCT biotinylated antibody	NBP1-79065B	Novus Biologicals
Mouse IgG1 Isotype control	MAB002	R&D system
Rat IgG1 Isotype control	14-4301-82	Invitrogen
Glucose oxidase	65C-CE0110	Fitzgerald
Human IL3 protein	203-IL-050	R&D system
Human IL6 protein	206-IL-010	R&D system
Human PCT protein	9607-PN-050	R&D system

Table S3. Clinical information on patient samples.

		Septic patients	COVID-19 patients	Healthy donors
**Number**		35	20	35
**Age**				
	Median	65	65	54
	Range	38 – 88	37 – 79	30 – 70
**Sex**				
	Male	27 (77%)	14 (70%)	22 (63%)
	Female	8 (23%)	6 (30%)	13 (37%)

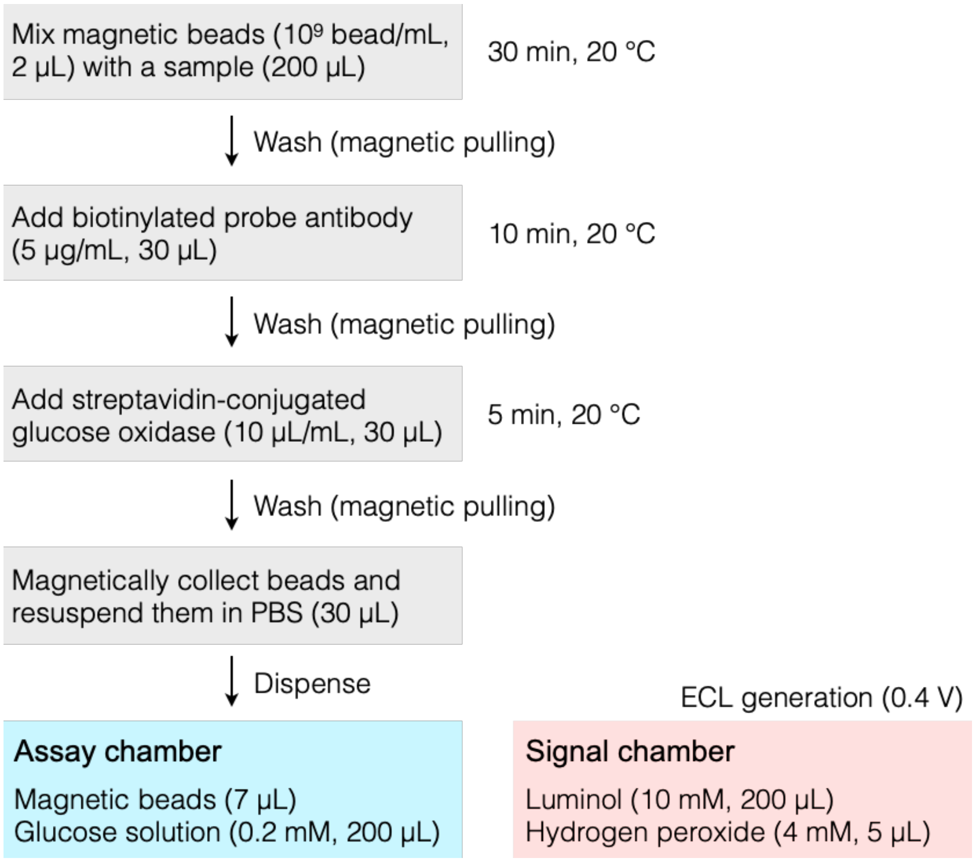

Figure S1. Detailed assay workflow.

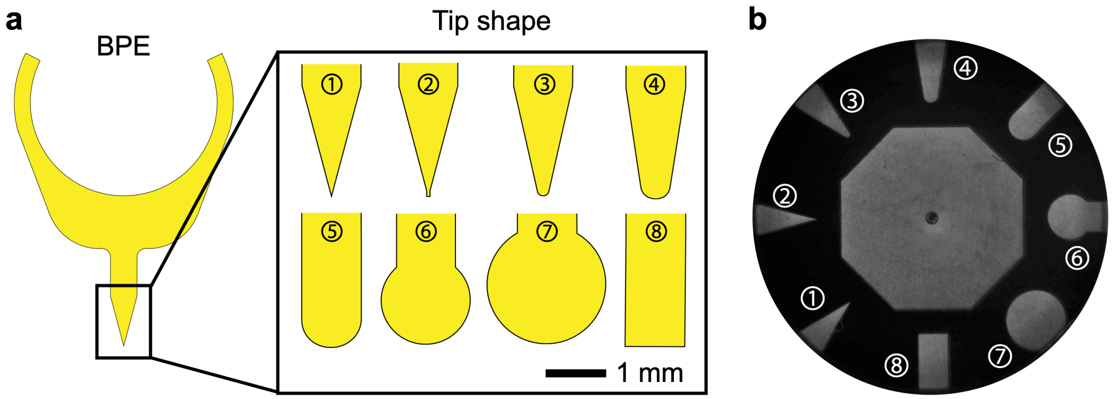

Figure S2. Various BPE designs were tested for ECL generation. a, The shape of signal electrodes was changed to find the optimal geometry for high ECL signal. The distances from each electrode's tip to the cathode were kept the same. **b,** Image of signal electrodes taken by a camera.

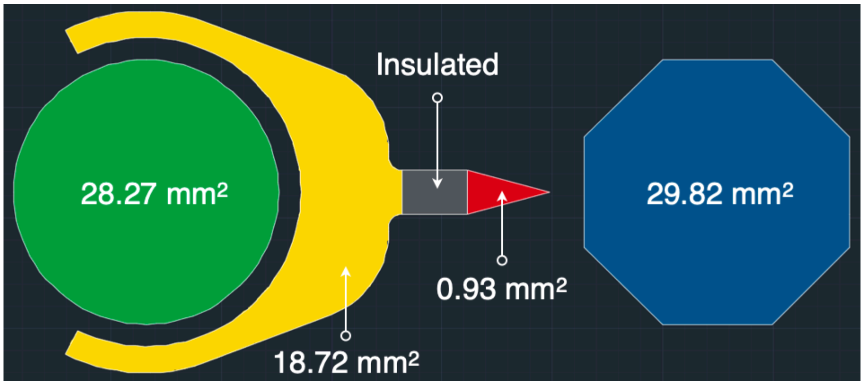

Figure S3. Electrode layout in the ECLipse chip. Green, an anode in the assay chamber; yellow, a cathodic portion of BPE in the assay chamber; gray, an insulated section of BPE; red, the anodic part of BPE in the signal chamber; blue, a cathode in the signal chamber.

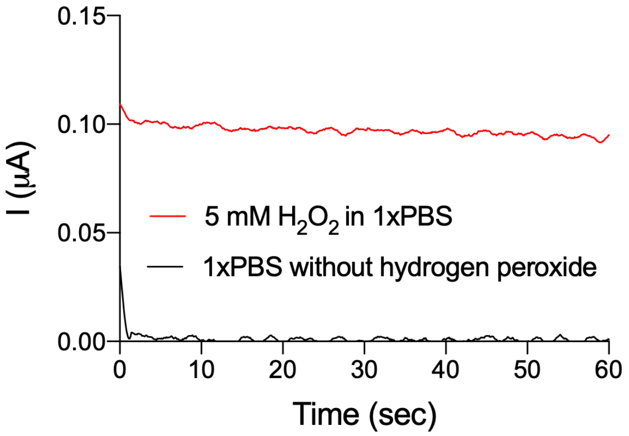

Figure S4. Oxidation of H_2O_2. Chronoamperometry confirmed that H_2O_2 was oxidized on a gold electrode at the applied potential of 0.04 V (red line). Without H_2O_2, no electrical current was measured (black line).

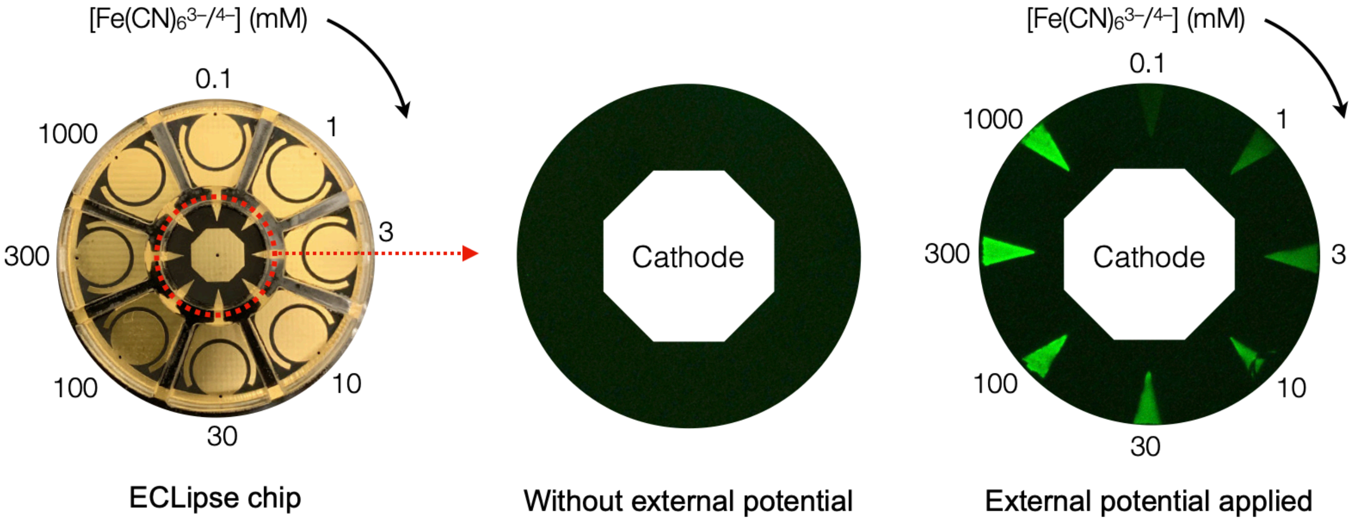

Figure S5. Optical images of an ECLipse chip. (Left) The assay chambers in the chip were loaded with samples containing varying amounts of $Fe(CN)_6^{3-/4-}$ for redox reaction. **(Middle)** Without the electrical potential applied, no ECL signal was observed in the signal chamber. **(Right)** An ECL signal was generated on BPE's triangular tip when the potential was applied. The signal intensity was proportional to $Fe(CN)_6^{3-/4-}$ concentration in the assay chamber.

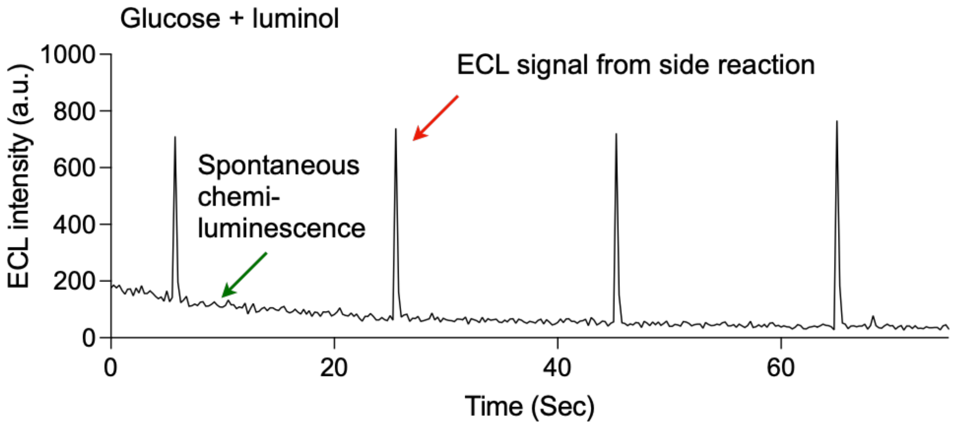

Figure S6. Background signal in the conventional ECL assay. Mixing two components, glucose (0.2 mM, 45 μL) and luminol (25 μM, 5 μL), generated two types of background signals from a side reaction: i) decaying chemiluminescence without electrical potential applied and ii) ECL when the electrical potential was applied. Note that a complete ECL reaction required a mixture of glucose, luminol, and H_2O_2 in the conventional one-pot assay.

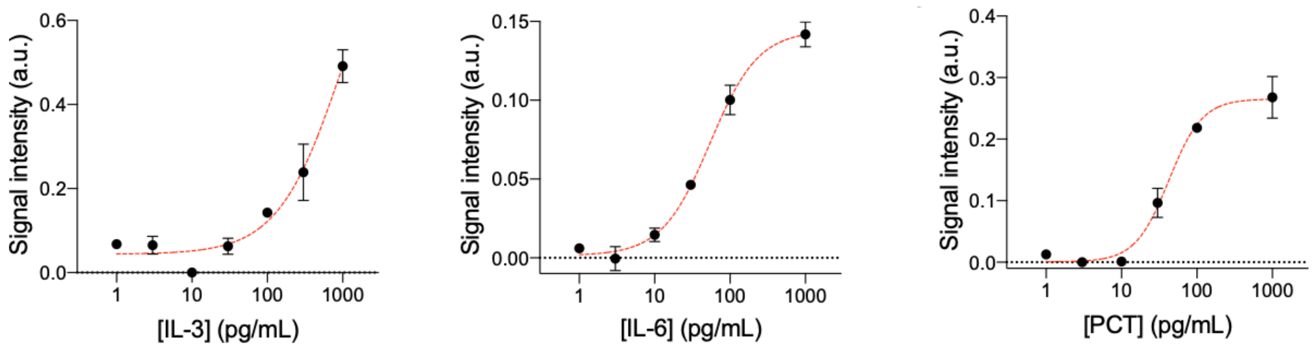

Figure S7. Titration curves of IL-3, IL-6, and PCT by ELISA. Varying amounts of protein targets were spiked into human plasma and quantified by ELISA. The limits of detection were 100 pg/mL (IL-3), 19.6 pg/mL (IL-6), and 28.7 pg/mL (PCT). Data are displayed as mean ± s.d. from duplicate measurements.

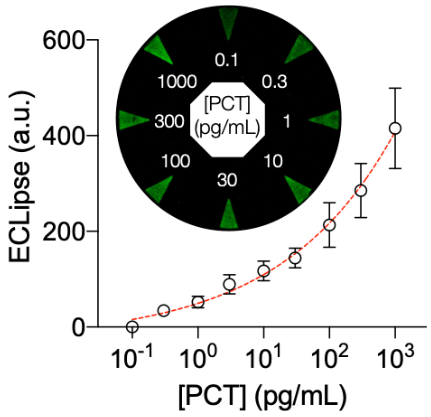

Figure S8. PCT titration by ECLipse. Human plasma samples were spiked with varying amounts of PCT and assayed by ECLipse. **(Inset)** Image of an ECLipse chip when samples with different PCT concentrations were measured. The limit of detection was 230 fg/mL. Data are displayed as mean ± s.d. from triplicate measurements.

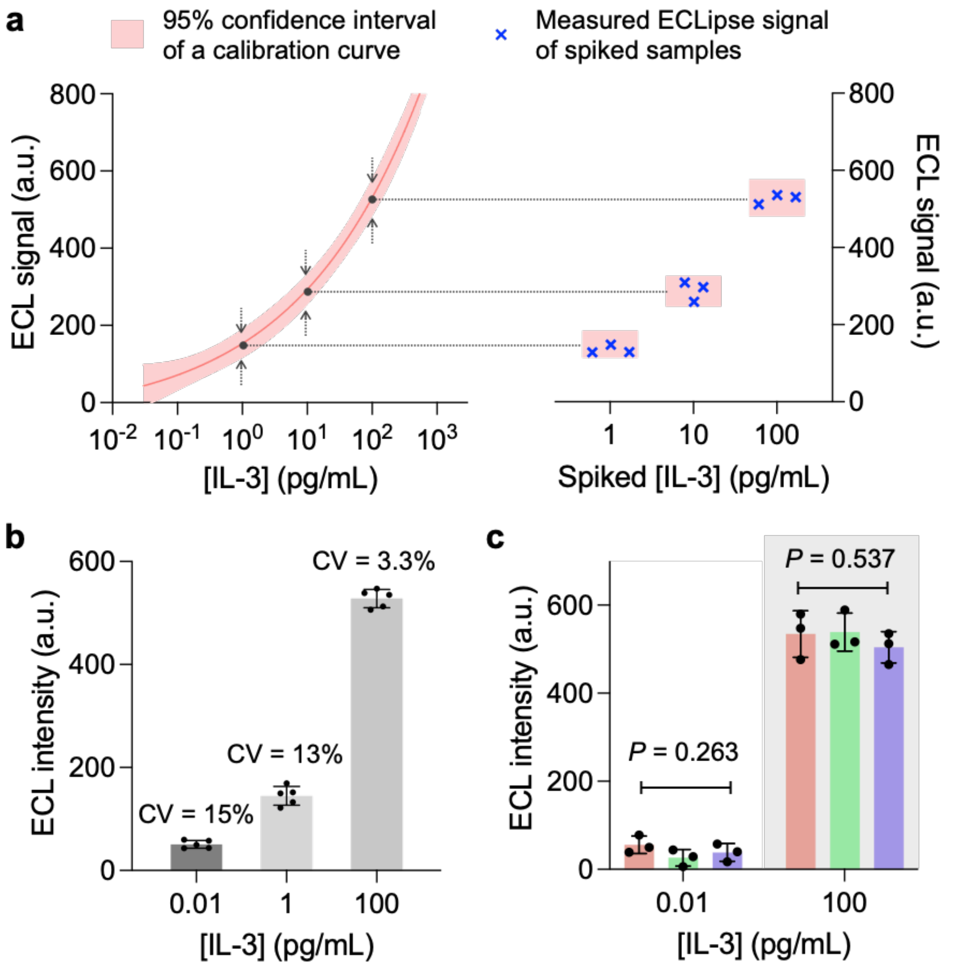

Figure S9. Characterization of the ECLipse assay. (a) Analytical accuracy. Spiked-in samples ([IL-3] = 1, 10, 100 pg/mL) were assayed by ECLipse, and the target concentrations were estimated from the pre-generated calibration curve (left). For a given spiked-in sample, the measured ECLipse value was within the 95% confidence interval (CI) of the calibration curve (right). **(b)** Intra-assay variations. The same samples were measured repeatedly ($n = 5$) in a given run. The relative coefficient of variation was ≤15%. **(c)** Inter-assay variations. Standard samples were measured at different times. For a given sample, the ECLipse results were statistically non-different among runs (one-way ANOVA).

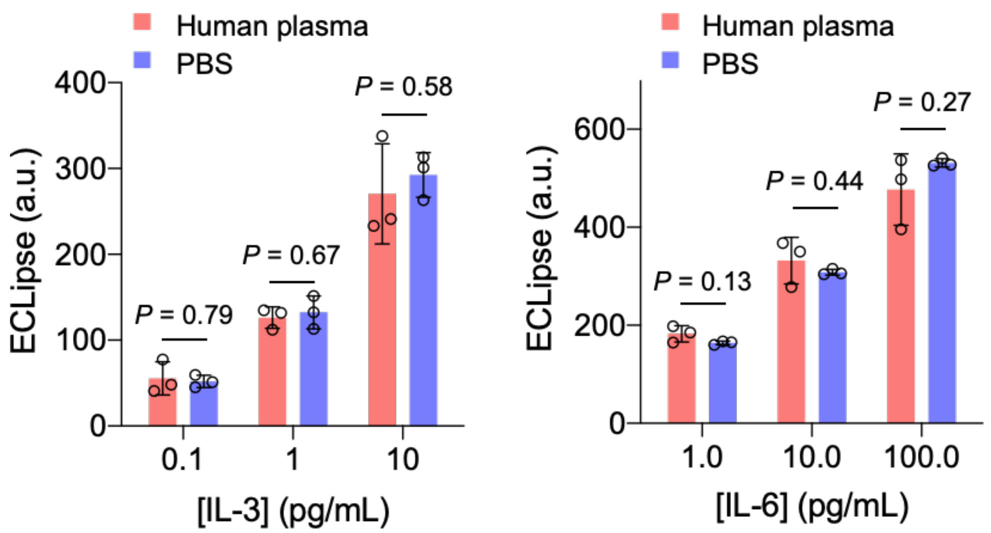

Figure S10. Signal comparison between different matrices. Test samples were prepared by spiking in target proteins in pure buffer (PBS) and human plasma. Measured ECLipse signals were statistically non-different between these two types of samples (unpaired two-sided *t*-test). For plasma samples, the signal from innate target proteins was subtracted. Data are displayed as mean ± s.d. from triplicate measurements.

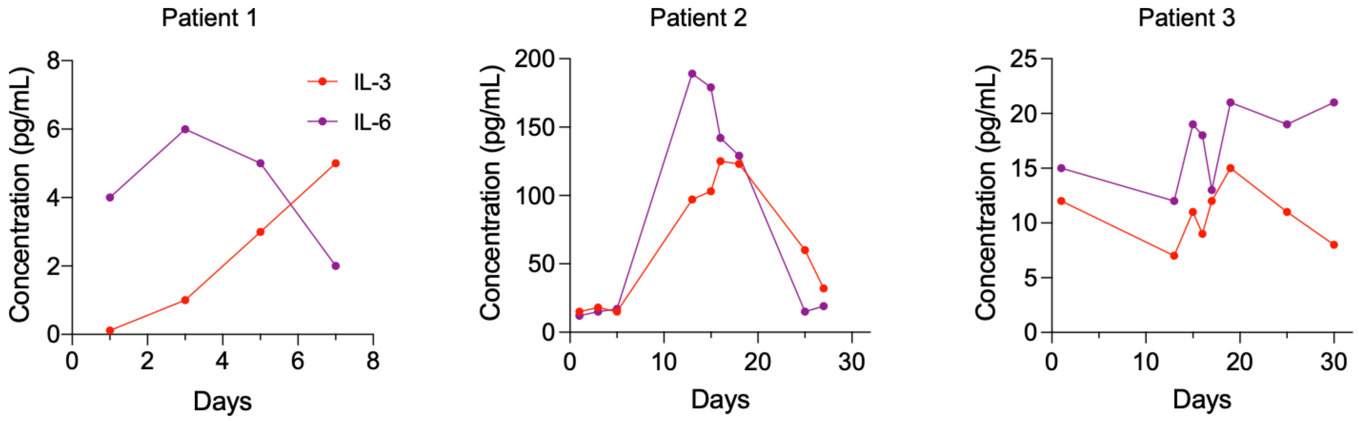

Figure S11. Longitudinal monitoring of IL-3 and IL-6 in sepsis patients. IL-3 and IL-6 concentrations in plasma were measured by ECLipse.

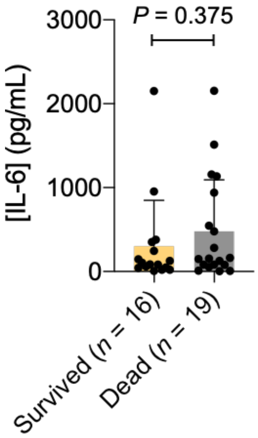

Figure S12. IL-6 concentration of patients at the sepsis onset. IL-6 levels showed no significant difference (*P* = 0.375, two-sided *t*-test) between survived and non-survived cohorts.
